# CD40 in coronary artery disease: a matter of macrophages?

**DOI:** 10.1007/s00395-016-0554-5

**Published:** 2016-05-04

**Authors:** Matthijs F. Jansen, Maurits R. Hollander, Niels van Royen, Anton J. Horrevoets, Esther Lutgens

**Affiliations:** Department of Molecular Cell Biology and Immunology, VU University Medical Centre, Amsterdam, The Netherlands; Department of Medical Biochemistry, Academic Medical Centre, Meibergdreef 15, 1105AZ Amsterdam, The Netherlands; Department of Cardiology, VU University Medical Center, Amsterdam, The Netherlands; Institute for Cardiovascular Prevention (IPEK), Ludwig Maximilians University, Munich, Germany

**Keywords:** CD40, Macrophage, Atherosclerosis, Arteriogenesis, Neointima formation, Ischemic heart disease

## Abstract

Coronary artery disease (CAD), also known as ischemic heart disease (IHD), is the leading cause of mortality in the western world, with developing countries showing a similar trend. With the increased understanding of the role of the immune system and inflammation in coronary artery disease, it was shown that macrophages play a major role in this disease. Costimulatory molecules are important regulators of inflammation, and especially, the CD40L-CD40 axis is of importance in the pathogenesis of cardiovascular disease. Although it was shown that CD40 can mediate macrophage function, its exact role in macrophage biology has not gained much attention in cardiovascular disease. Therefore, the goal of this review is to give an overview on the role of macrophage-specific CD40 in cardiovascular disease, with a focus on coronary artery disease. We will discuss the function of CD40 on the macrophage and its (proposed) role in the reduction of atherosclerosis, the reduction of neointima formation, and the stimulation of arteriogenesis.

## Introduction

The TNF receptor superfamily member 5 (TNFRSF5), or CD40, is a costimulatory molecule that was originally discovered on B-cells and other antigen presenting cells (APCs) [110]. CD40 is activated by its ligand, CD40L(TNFSF5) [89]. CD40 is expressed on a multitude of immune cells and non-immune cells, with functions varying per cell type [21, 41]. In B-cells, CD40 ligation induces T-cell-dependent immunoglobulin class switching [42], memory B-cell development [48], and germinal center formation [71, 79]. In dendritic cells, CD40 ligation induces more effective antigen presentation [17, 115, 124], enhances T-cell stimulatory capacity, and induces production of several inflammatory cytokines and chemokines [18]. It was discovered recently that T-cells also express CD40 but not much is known about its function. T-cell CD40 seems to mediate CD8+ T-cell memory [12], can contribute to T-cell activation [107], and is associated with autoimmune disease [142, 143]. On monocytes, CD40 stimulation induces the production of inflammatory cytokines and chemokines [75], and matrix metalloproteinases [38] and, similar to CD40 on dendritic cells, induces more potent antigen presentation [17, 115, 124]. The effects of CD40 on macrophages will be described in detail below.

In the 1990s, it was discovered that blocking CD40L limits atherosclerosis [91, 93, 128] and induces a stable plaque phenotype in mice [90]. Thereafter, it was shown that knocking out CD40, the receptor for CD40L, induced a similar phenotype [92]. Our laboratories have shown the importance of CD40 on hematopoietic cells, and macrophages in particular. We showed that a deficiency of hematopoietic CD40 decreased atherosclerosis and induced plaque stabilization in CD40 knock-out mice [92]. Macrophages of these mice were of the regulatory M2 phenotype. We also showed that the antiarteriogenic protein galectin-2 shifts proarteriogenic, CD40-negative macrophages into proinflammatory, and CD40-positive macrophages, resulting in compromised arteriogenesis [158]. We identified galectin-2 to be highly expressed in monocytes of human chronic total coronary occlusion (CTO) patients with a poor collateral network, compared with CTO patients with a well-developed collateral network [145]. These findings, in combination with the large overlap between functions of CD40 and macrophages in cardiovascular disease, suggest an important role of macrophage-specific CD40 in cardiovascular disease. Specific inhibition of macrophage CD40 might act as a “double-edged sword” by inhibiting atherosclerosis and stimulating arteriogenesis, resulting in a reduced ischemic burden without interfering in adaptive immunity.

## Macrophages in cardiovascular disease

Monocytes and macrophages largely contribute to the pathophysiology of cardiovascular diseases, for example, in atherosclerosis [4, 37, 57, 62, 120, 164] and arteriogenesis [55, 58]. Both monocytes and macrophages can, at the extremes, be divided in a proinflammatory phenotype and a healing phenotype. The interplay and balance between these two phenotypes have shown to be of importance in, for example, atherosclerosis [25, 29, 130] and myocardial infarction [37, 154]. In murine monocytes, the proinflammatory phenotype is defined as Ly6C high, while the healing phenotype is defined as Ly6C low [159]. Ly6C high monocytosis is regarded as one of the first steps in the inflammatory response in atherosclerosis, as Ly6C high monocytes activate endothelium, infiltrate into the intima, and become lesional macrophages. Furthermore, in atherosclerosis models, such as the apolipoprotein (ApoE) deficient mouse, hypercholesterolemia is associated with Ly6C high monocytosis. Inhibition of the Ly6C high monocytosis abolishes atherosclerosis in hypercholesterolemic mice [26, 87, 136]. In humans proinflammatory, or classical, monocytes are generally defined as CD14++/CD16−, while the healing, or non-classical, phenotype is defined as CD14+/CD16++ [166]. An intermediate, CD14++/CD16+ population can also be observed in humans [99, 153]. In concurrence with the animal model described above, in humans, CD14++/CD16− monocytosis is associated with atherosclerosis and is an independent predictor of cardiovascular events [61, 123]. In macrophages, the phenotypic spectrum is defined by the proinflammatory M1 macrophages that are induced by T-helper 1 cytokines, and by M2 macrophages that are induced by Th-2 cytokines. The M2 macrophages can be subclassified into wound healing (M2a), regulatory (M2b, M2c), and M2d subtypes [24, 25, 104]. In addition, atherosclerosis-associated macrophage phenotypes have been discovered, i.e., M(Hb), Mox, Mhem, and M4 macrophages [24, 25]. Consequently, a large number of macrophage phenotypic markers have evokes (reviewed by Mosser et al. [104]. and Colin et al. [25]). While CD40 is not mentioned in these reviews, it has proved to be a distinctive marker for M1 macrophages [148, 149]. In experimental atherosclerosis models, the M1 and the plaque specific M4 subtypes are proinflammatory and proatherogenic and seem to cause a vulnerable plaque phenotype. The M2 phenotype and the plaque specific M(hb) and Mhem are anti-inflammatory and antiatherogenic. The role of the Mox phenotype is currently not well understood [25, 29]. The role of the M2 subtypes has not been defined further yet. In arteriogenesis in animal models, the shift toward M2 phenotype (no data on subtypes) improves arteriogenesis and reduces ischemia [51, 138, 140]. In humans, M1 macrophages are associated with plaque instability, both in ischemic stroke and in myocardial infarction [23, 81, 134]. A word of caution should be added, regarding the dichotomous distinction between M1 and M2 macrophages, as these terms are increasingly discouraged by immunologists. In vivo, a wide range of M1- and M2-like macrophages can be distinguished. While older studies use the M1/M2 nomenclature, it is now encouraged to use multiple markers to describe the macrophage phenotype [47]. However, this review will still use the M1/M2 nomenclature, as the studies described all use this nomenclature.

## CD40 in clinical disease

Cardiovascular diseases share many traits of their pathophysiology with other autoimmune diseases, such as rheumatoid arthritis [68], systemic sclerosis [16], systemic lupus erythematosus [3], or inflammatory bowel disease [49, 72, 139]. CD40 and CD40L were shown to have a pivotal role in these diseases [27, 33, 96, 114]. Interestingly, higher sCD40L levels in patients with Crohn’s disease even predicted thicker intima and media in their carotid arteries [72]. In cardiovascular diseases, blocking CD40 signaling has never been tested in clinical trials. Anta- and agonistic CD40 antibodies, however, have been tested in other chronic inflammatory diseases and cancer. Below is a brief overview of these trials and the effectiveness and side effects of blocking or activating CD40 signaling.

In 1999, the first clinical trials started using an anti-CD40L antibody as a treatment for systemic lupus erythematosus, lupus glomerulonephritis, and immune thrombocytopenic purpura. The anti-CD40L antibody treatment showed an improvement in the number of platelets in immune thrombocytopenic purpura but did not improve performance scores in systemic lupus erythematosus [11, 28, 69, 78]. Blocking CD40L was effective in lupus glomerulonephritis, where it markedly reduced hematuria. However, this trial was ended prematurely because of the high incidence of thromboembolic events [11]. These thromboembolic events are most likely due to the fact that CD40L is also present on platelets, as inhibition of CD40L causes platelet aggregates to become unstable and to embolize [5]. Since the anti-CD40L antibody treatment was deemed unsafe, attention was shifted toward the CD40 molecule.

Dacetuzumab is a humanized anti-CD40 agonistic mAb, which triggers CD40-mediated signaling in various cell types [53]. It has been tested in several hematologic malignancies. In a phase I single agent study of patients with relapsed B-cell non-Hodgkin’s lymphoma, six of 50 patients had objective response, and an additional thirteen had documented stable disease [2]. For relapsed chronic lymphocytic leukemia, a phase I single agent study was performed. In this study, none of the patients achieved an objective response; however, five out of twelve patients showed stable disease [43]. In refectory multiple myeloma, single agent therapy with dacetuzumab showed no objective response in a phase I study [60]. In refractory diffuse large B-cell lymphoma, a phase I study initially showed promising results of dacetuzumab as a single agent, with objective responses in four of 46 patients and 13 cases of stable disease [30]. However, a follow-up study showed no benefit of dacetuzumab on top of the existing last-resort chemotherapy [36]. In these studies, about two-thirds of patient’s experienced adverse events classified as grade 1–2 out of 4 among them were fatigue, headache, pyrexia, chills, nausea, anemia, thrombocytopenia, and hypotension. Non-infectious eye disorders, including conjunctivitis and ocular hyperemia, were also seen. A few grade 3 adverse events were seen, including malignant neoplasm progression, severe anemia, pleural effusion, thrombocytopenia, and severe infections. Some grade 4 events were seen, including aseptic meningitis and hyperviscosity syndrome [2, 30, 36, 43, 60].

Lucatumumab (or HCD122) is a fully humanized antagonistic antibody against CD40 and exerts its primary function through opsonization followed by antibody-dependent cell-mediated cytotoxicity (ADCC) [53]. It has been tested in a single-agent phase I study of relapsed chronic lymphocytic leukemia [15]. Here, one of 26 patients had a partial response and sixteen had a stable disease. For relapsed or refractory multiple myeloma, a single agent phase I study was performed in 28 patients. Twelve patients had a stable disease after treatment and one patient maintained a partial response for up to 8 months [8]. A phase I/IIa study was performed for advanced non-Hodgkin and Hodgkin lymphoma in 111 patients. In this study, the overall response rate by computed tomography among patients was 33 % [35]. Overall, most adverse events observed in these studies were mild to moderate, including neutropenia, thrombocytopenia, fatigue, headache, chills, fever, and nausea, cytokine release syndrome symptoms (mostly grades 1–2, sometimes grades 3–4), non-infectious ocular inflammation, and elevated hepatic enzymes. A few serious adverse events were seen, including dyspnea, pyrexia elevated liver enzymes and infections. One death due to severe sepsis was reported [8, 15, 35].

An antagonistic anti-CD40 antibody, ch5D12, has been tested in 18 patients with mild to moderate Crohn’s disease. Based on Crohn’s Disease Activity Index (CDAI) scores, the overall response rate was 70 %, remission was achieved in 22 % of patients, and ch5D12 was well tolerated. Described side effects were mild, including headache, muscle aches, or joint pains [70].

While the side effects are described as overall mild to modest in these papers, this is in comparison with the conventional therapy for cancer or Crohn’s disease. However, in chronic use for ischemic heart diseases, the side effects would be more damaging than beneficial. Since blocking CD40 or CD40L systemically is not suitable for the treatment of ischemic heart diseases because of predicted severe side effects, such as immune suppression, targeting CD40(L) downstream targets and selected CD40 effector cells may be a preferred strategy.

## CD40 and macrophages

The findings described above, in combination with the large overlap between functions of CD40 and macrophages in cardiovascular disease suggest an important role of macrophage-specific CD40 in cardiovascular disease. Unfortunately, there is not much data available on signaling pathways elicited by CD40 in macrophages or macrophage subtypes. Since CD40 is expressed mostly on M1 macrophages [148, 149], it can be assumed that CD40 signaling is predominantly active in M1 macrophages. Ligation of CD40 on macrophages induces a more potent antigen presentation, with the upregulation of MHC class II, costimulatory molecules CD80, CD86, and CD40 itself [135]. Furthermore, ligation of CD40 stimulates the production of proinflammatory cytokines and chemokines by macrophages, including TNFα, IL-1 (α and β), IL-6, IL-8, IL-12, CCL 2, 3, 4, and 5 [135]. CD40 also induces several other molecules, such as matrix metalloproteinases, nitric oxide, and possibly iNOS (NOS2) and COX-2. In addition, ligation of CD40 on near-apoptotic cells rescues them from apoptosis [118, 135].

However, since these studies are all performed in vitro, caution is needed when interpreting these interactions as the response CD40 induces in macrophages seems highly dependent on the environment. In vitro, the presence of IL-4 or IL-10 has been shown to induce quite different results in downstream CD40 signaling in macrophages. IL-4 blocks CD40 mediated rescue from apoptosis, while IL-10 does not [117]. IL-10 significantly inhibited CD40-induced activation of the ERK, p38 MAPK, and NF-κB pathways, whereas IL-4 only affects the ERK pathway [66]. CD40-CD40L interaction induces ROS production, the synthesis of ICAM1, and activation of stress response proteins (p38 MAP kinase and HSP27) only in the presence of hypoxia, indicating that CD40(L) mediates the induction of oxidative stress in these cells [20]. When comparing a model of an atherosclerotic plaque in mice [90] to a model of lung fibrosis in mice [1], opposite reactions to the same stimuli are seen due to the influence of tissue environment on CD40 signaling. In both models, inhibition of CD40-CD40L signaling results in a downregulation of inflammation. However, in atherosclerosis, anti-CD40L antibody treatment resulted in the upregulation of TGF-beta on macrophages, whereas in irradiation-induced lung injury, it caused downregulation of TGF-beta.

Environmental differences like these are important in tumor progression [111, 113], but there is also indirect evidence that these environmental differences play a role in other diseases. For example, the lack of apoptosis of macrophages inside the atheromatous plaque is thought to aggravate atherosclerosis [88, 146]. It was shown that IL-4 blocked CD40-mediated rescue from apoptosis, while IL-10 does not [117]. Though never proven, one could imagine that the plaque environment alters CD40 signaling, and thus affects atherosclerosis.

## TNF-receptor-associated factors

**Fig. 1:**
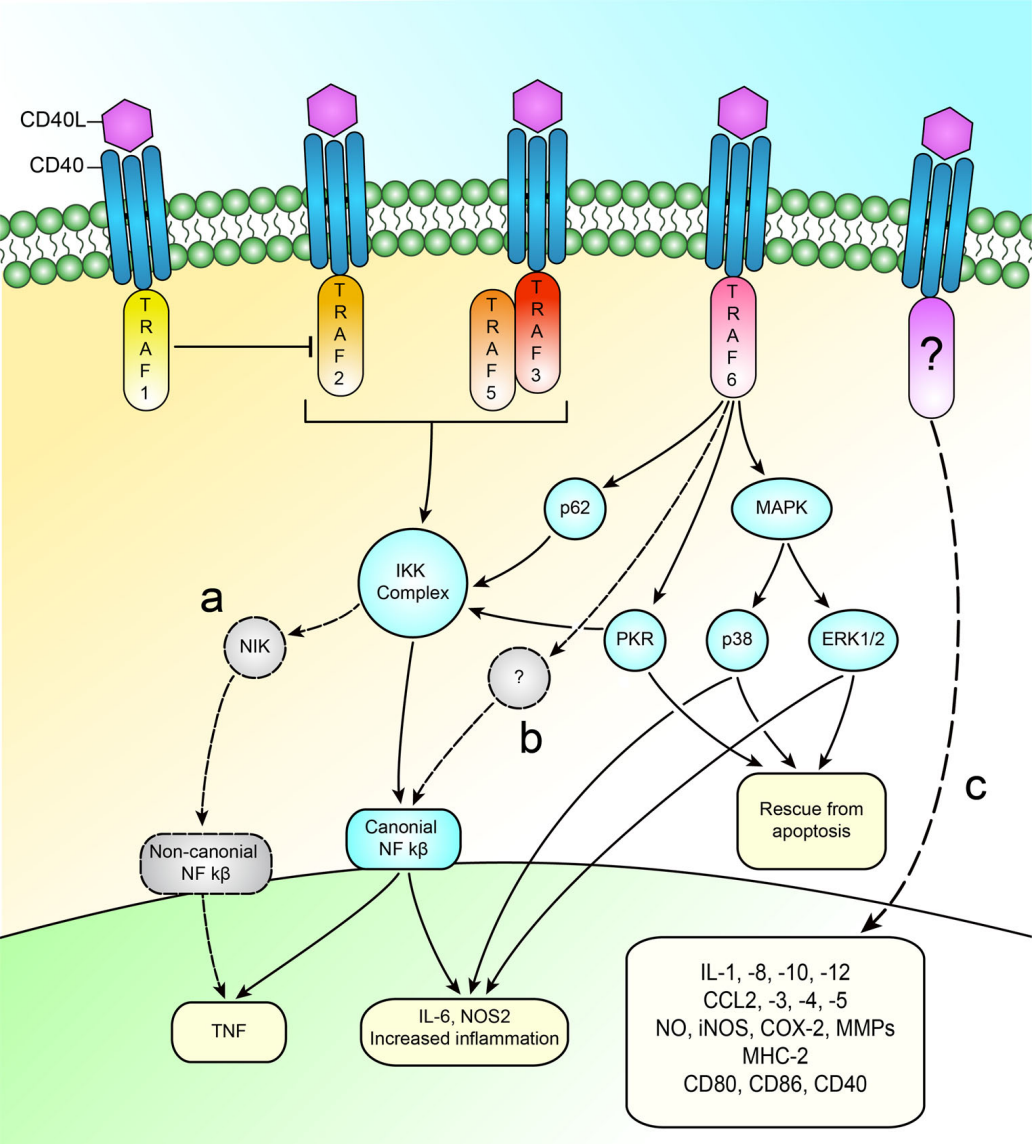
Overview of the main pathways involved in CD40-TRAF signaling. **a** TRAF 2/3/5 induces about 40 % of TNF production; however, they do not influence (canonical) NF-κB. Therefore, an alternative pathway must be present. Based on data from B-cells, this is most likely the non-canonical NF-κB pathway. **b** TRAF 2/3/5 is required for the IKK-complex; however, they do not influence (canonical) NF-κB. Therefore, an alternative pathway is likely present. **c** Much of the downstream targets of CD40-TRAF signaling still need to be uncovered. Of the majority of molecules produces by macrophages in response to CD40 signaling, no TRAF molecule or further pathway has been determined

As CD40 does not have the ability to initiate its own intrinsic activity but requires adaptor molecules, it is important to note the functions of these molecules, with a focus on their functions in macrophages. The TNF-receptor-associated factor (TRAF)-family of proteins can bind to the cytoplasmic tail of CD40 and subsequently recruit kinases and other effector proteins [127]. All TRAF-family members, with the exception of TRAF5, are ubiquitously expressed, suggesting that they may perform significant physiological and cellular functions in multiple organs and cell types. The TRAF-family members can be recruited by a variety of receptors, among which CD40. CD40 can, after activation by CD40L, recruit several different TRAF-family members. Depending on the TRAF-molecule activated, different transduction cascades are induced. These transduction cascades activated by the different TRAFs can have opposite effects. Unfortunately, what regulates the recruitment of a certain TRAF-family member to CD40 remains unclear. Of the seven known TRAF-family members, CD40 can bind five (TRAFs 1, 2, 3, 5, and 6) [119, 155]. CD40 contains three binding sites, one for the TRAF 1, 2, and 3, one for TRAF6 [119] and a secondary TRAF2 binding site. There is some conflicting evidence whether or not TRAF 5 can bind directly to the binding site of TRAF 1, 2, and 3 [67] or that it indirectly binds to it via an heterodimeric complex with TRAF3 [119, 161]. Below is a brief description of the different functions of the TRAF proteins when CD40 ligation occurs. A graphical overview of the main pathways involved in CD40-TRAF signaling is shown in Fig. 1. As TRAF4 and TRAF7 do not bind to CD40, they will not be discussed.

### TRAF1

TRAF1, like all TRAFs, can be recruited to a variety of TNRF receptor members, including CD40, TNFR I/II, and RANK [64]. In general, it is thought that TRAF1 has a role as a negative regulator of signaling in TNF receptors. This is displayed in TRAF1 knock-out models, where T-cells respond in a hyper proliferative manner in response to stimulation [82, 162]. However, the possible role as negative regulator has not yet been researched in macrophages. On the CD40 protein, TRAF1 shares a binding site with TRAF 2 and 3 and binds only weakly to this site [119]. No functional data are known about CD40-TRAF1 signaling on macrophages. However, ligation of CD40 on T- and B-cells that were deficient in TRAF1 did not show any differences compared with normal T- and B-cells [82, 162]. However, in cell cultures of dendritic or HeLA cells, TRAF1 negatively regulates CD40-TRAF2 signaling [6, 40]. Of interest, this review is a study by Missiou et al., which showed that TRAF1 deficiency reduces atherosclerosis by limiting the adhesion of monocytes to the vessel wall, suggesting that TRAF1 plays a role in monocyte adhesion [97]. However, since TRAF1 interacts with more receptors that just CD40, it is not known if this inhibition of adhesion was solely due to the lack of CD40-TRAF1 signaling.

### TRAF2

The general functions of TRAF2 are very broad. TRAF2 is required in T- and B-cell signaling and inflammatory responses, but it is also required for organogenesis and cell survival [7]. TRAF2 activates the canonical NF-κB signaling pathways [103] and also seems to be a negative regulator of the non-canonical NF-κB signaling pathways, as TRAF2 deficiency results in hyperreactivity of this pathway [7]. On the CD40 protein, TRAF2 shares its binding site with TRAF1 and 3. It binds strongly and directly to this binding site [119]. TRAF2 seems to require an intact lipid raft to function, as CD40-TRAF2 signaling on dendritic cells is largely absent when disturbing the lipid raft [147]. On the macrophage, some evidence points toward the requirement of TRAF2 expression and subsequent degradation after stimulation with CD40L for the differentiation of monocytes into fully functional macrophages [34], suggesting that TRAF2 has a low concentration in macrophages. A defective TRAF2, 3, and 5 binding site on CD40 in macrophages does not completely impair the inflammatory response to CD40L; however, it does result in a lower TNF production (ca. 40 % of total production). Furthermore, CD40-TRAF2/3/5 deficiency leads to an inability to activate the IKK complex. However, this defect has no consequences for NF-kB activation, IL-6 production, or ERK1/2 activation [65, 106].

### TRAF3

The function of TRAF3 has long been a mystery, as researching it proved difficult. TRAF3 knockout mice have a relatively normal gestation period; however, soon after birth, the mice die prematurely within two weeks of age with symptoms, including stunted growth and progressive hypoglycemia, hypercortisolemia, and leukopenia [54]. Relatively, recently, it was discovered that TRAF3 is a powerful negative regulator of the non-canonical NF-κB signaling pathways and a modulator of viral immunity [54]. On the CD40 molecule, as mentioned before, TRAF3 shares its binding site with TRAF1 and 2. It binds strongly and directly to this binding site [119]. TRAF3, like TRAF2, also seems to require an intact lipid raft to function; however, the effect of disturbing the lipid raft is not as large as with TRAF2 [147]. On macrophages, TRAF3 is suggested to only be present in low concentrations. Monocytes showed a strongly immunoreactivity for TRAF3, but macrophages typically contained little or no TRAF3 immunoreactivity [77].

### TRAF5

TRAF5 is highly similar to TRAF2, in both structure and function. However, whereas TRAF2 is expressed ubiquitously, TRAF5 expression is only found at significant levels in lung, thymus, spleen, and kidney and at lower levels in the brain and liver [7]. A sole TRAF5 deficiency has interesting consequences. TRAF5 deficiency accelerates atherogenesis in a mice model by promoting the rolling and adhesion of inflammatory cells and macrophage LDL uptake, which might contribute to foam cell formation [98], suggesting an anti-inflammatory, antiatherogenic function of TRAF5. It is under discussion whether TRAF5 directly [67] or indirectly binds to CD40 via hetero-oligomers with TRAF3 [119, 161].

### TRAF6

Generation of TRAF6 deficient mice revealed that TRAF6 plays crucial roles in several important processes and that other TRAFs cannot compensate for loss of TRAF6. These functions are extremely broad, including osteoclastogenesis, lymph node organogenesis, thymic selection, and central tolerance. It is essential for IL-1 signaling and required for most TLR-receptor signaling [63, 151]. Furthermore, TRAF6 mediates antiviral responses triggered by cytosolic viral DNA and RNA in a way that differs from that associated with TLR signaling [76]. On the CD40 protein, TRAF6 has a separate binding site, to which it weakly binds [119]. Upon binding, TRAF6 is required for the maturation of dendritic cells, the affinity maturation of immunoglobulins, and optimal function of CD8+ T-cell homeostasis and memory development [63, 151]. In monocytes and macrophages, TRAF6 is responsible for the most downstream actions of CD40. It is required for TNF production, IL6 production, NF-κB activation (through p62 [129]), ERK activity, IKK activation, PKR phosphorylation [106], and upregulation of NOS2 (in cooperation with TNF) [118]. Defective CD40-TRAF6 signaling has some interesting results in monocytes and macrophages. This deficiency induces the polarization of macrophages toward an anti-inflammatory regulatory M2 signature, induces a reduced blood count of Ly6C high monocytes, and results in impaired recruitment of Ly6C high monocytes to the arterial wall. This defective signaling also results in a marked reduction of atherosclerosis in a mouse model [22, 92]. Recently, a small molecule CD40-TRAF6 inhibitor was developed. This compound showed significant survival benefits in sepsis and peritonitis in mice, with little side effects [163]. Furthermore, in a diet-induced obesity model, it was shown that the small molecule inhibitor reduced insulin resistance and, most important for this review, reduced the accumulation of immune cells to the adipose tissue and by skewing of the immune response toward a more anti-inflammatory profile [22, 144].

## Atherosclerosis

In the pathophysiology of coronary artery disease, macrophage-specific CD40 plays a role in four major processes of the disease, atherosclerosis, neointima formation, angiogenesis, and arteriogenesis. Below the role of CD40 and macrophage-specific CD40 in these four processes will be described.

In atherosclerosis, atheromatous plaques develop due to the accumulation of apolipoprotein B containing lipoproteins in the inner lining of large- and medium-sized arteries [101]. When the coronary vasculature is affected, this can lead to ischemic heart disease. Ischemic heart disease is an important determinant of morbidity and mortality in developed countries, and is soon to attain this status worldwide [105]. As the atheromatous plaques develop, they grow larger and can restrict the vessel lumen resulting in symptoms, such as angina pectoris or claudicato intermittens, depending on the arterial bed affected. When atherosclerosis progresses, there is an increase in the incidence of the acute and most damaging complications, such as myocardial infarctions or ischemic cerebrovascular events. These acute complications of atherosclerosis are caused through either the rupture of a vulnerable plaque or the erosion of the endothelial layer [85, 108].

In the 1990s, it was discovered that inhibition of CD40 signaling by blocking CD40L limits the evolution of established atherosclerosis in mice [91, 93, 128]. In concurrence with these findings, it was shown that both early and delayed anti-CD40L antibody treatment induced a stable plaque phenotype [90]. Furthermore, it was shown that platelet CD40L mediates thrombotic and inflammatory processes in atherosclerosis [86]. Moreover, Leroyer et al. showed that microparticles isolated from human atherosclerotic lesions express CD40L, stimulate endothelial cell proliferation after CD4 ligation, and promote in vivo angiogenesis. The majority of these microparticles (93 %) were of macrophage origin, and therefore, microparticles released by macrophages could represent a major determinant of intraplaque neovascularization and, thus, plaque vulnerability [83]. In humans, CD40L predicts cardiovascular events. In unstable coronary artery disease, expression of CD40L on platelets and serum soluble CD40L levels are higher in patients compared with stable coronary disease or peripheral arterial disease [10, 45]. Furthermore, some studies show that sCD40L concentrations can predict clinical outcome in patients with acute coronary syndrome. However, other studies report no correlation between sCD40L levels and clinical outcome [112]. CD40L antibodies were tested clinically for other autoimmune diseases (see “CD40 in clinical disease”). However, trials were ended because of the high incidence of thromboembolic events [11]. These were most likely caused by to the fact that CD40L is also present on platelets, and inhibition of CD40L causes platelet aggregates to become unstable [5].

Thus, attention was shifted toward the CD40 protein. The involvement of CD40 signaling in atherosclerosis has been firmly established. In a mouse model that lacked both ApoE and CD40-TRAF6 signaling, atherosclerosis was abolished. Furthermore, the defective CD40 signaling induced a clinically favorable plaque phenotype, containing a high amount of fibrosis and a few inflammatory cells [92]. Furthermore, it was also shown that macrophage foam cell formation is highly dependent on CD40 in a study by Yuan et al. Soluble sCD40L significantly increased lipid deposition and foam cell formation. Disruption of the ligation between CD40 and CD40L either by small interfering RNA or by a blocking anti-CD40 antibody inhibited foam cell formation in response to sCD40L [160].Moreover, a meta-analysis of the rs1883832 CD40 SNP has shown a correlation between the C allele of this SNP and acute coronary syndrome in a Chinese population [157].

There is convincing evidence, suggesting a large role of CD40 on macrophages in atherosclerosis. The mouse knock-out model described above that lacked both ApoE and CD40-TRAF6 signaling displayed a reduced blood count of Ly6C high monocytes, an impaired recruitment of Ly6C high monocytes to the arterial wall, and polarization of macrophages toward an anti-inflammatory regulatory M2 signature. The reduction in atherosclerosis and the macrophage phenotype shift, both induced by defective CD40-TRAF6 signaling, is suggestive for a large role of CD40 on macrophages in atherosclerosis. In patients, CD40 on monocytes and macrophages is a marker for atherosclerosis, as shown by Bruemmer et al., who discovered that there is a direct association of CD40 expression on macrophages and smooth muscle cells and intimal thickness, suggesting a role in early plaque development [13]. In addition, CD40 on macrophages is associated with critical limb ischemia [14] and coronary artery calcification [137] in patients. Furthermore, patients with moderate hypercholesterolemia showed a significant increase in CD40 on monocytes (together with CD154 and P-selectin on platelets) compared with healthy subjects. A short-term therapy with an HMG-CoA reductase inhibitor significantly downregulated CD40 on monocytes [46, 150]. Unfortunately, a model detailing the effect on atherosclerosis by a specific deletion of CD40 on macrophages has not been published yet. On a side note, the difference described between males and females in the prevalence in cardiovascular diseases [141] might be in part due to a difference in CD40 on human macrophages. Androgens increase the expression of CD40 (among other atherosclerosis related genes) in male but not female macrophages, with functional consequences [109].

## Neointima formation

Essentially, all damage to the vascular wall results in an increase in intimal thickness, or neointima formation. This process is associated with luminal narrowing, causing major complications in humans after arterial intervention (e.g., balloon angioplasty and stenting). In neointima formation, vascular smooth muscle cells proliferate within the intima. This process has been the main target for treatment in the form of drug eluting stents. However, the immunological process driving smooth muscle cell proliferation has not been fully uncovered, though initial cytokine release from platelets and macrophages, and further aggravation through macrophage produced cytokines has been suggested [19, 74]. A role for CD8+ T-cells has also been shown, where the absence of CD8+ cells increases neointima formation. Conversely, the absence of CD4+ T-cells reduces neointima formation [31, 165].

In ApoE deficient mice in which denudation injury to the carotid arteries was induced, it was shown that blocking CD40L significantly reduced the exaggerated neointima formation, with a >50 % reduction in neointimal size and a 56 % reduction in neointimal macrophage content [84]. Moreover, it was shown that blocking CD40L-CD40 signaling but not CD40L-Mac-1 reduces neointima formation [152]. In other studies, it was shown that neointima formation after both carotid artery ligation and femoral artery denudation injury was reduced in CD40-deficient mice compared with wild-type mice. Furthermore, a significant decrease in the recruitment of neutrophils (at 3 and 7 days) and macrophages (at 7 and 21 days) into injured artery was shown [59, 132]. Some contradictory data exist as Remskar et al. showed that after carotid artery injury in CD40L knockout mice, the intimal thickening was increased 3-fold compared with the thickening in wild-type mice [122]. Furthermore, it was shown by Donners et al. that inhibition of CD40L signaling did not reduce neointima formation in mice. CD40-knock-out mice, however, did have reduced neointima formation in the same study, suggesting that CD40, but perhaps not CD40L, plays a large role in neointima formation [32].

Like in atherosclerosis, TRAF6 seems to be the key regulator of CD40 signaling in neointima formation and arterial remodeling. Donners et al. showed that in mice deficient for CD40-TRAF6 signaling, neointima volume was reduced by 83 %. Also like atherosclerosis, they showed an impaired recruitment of macrophages to the vessel wall [32]. Another study demonstrated that in rabbits where carotid artery damage was induced by balloon inflation, CD40-TRAF6 signaling deficiency inhibited intimal cell replication, macrophage infiltration, and proteoglycan accumulation [100]. The role of CD40-TRAF6 signaling on vascular wall cells was also shown to be of importance, cooperating with kinase TAK1 [131, 133]. These data suggest a possible strategy for preventing neointima formation and, thus, improve outcome after vascular intervention by interfering in the CD40, most likely CD40-TRAF6, signaling of macrophages. However, no indication of its effectiveness compared with drug eluting stents, the current clinical practice, has been made.

## Angiogenesis

Angiogenesis, a process seen in both health and disease, refers to the formation of new capillaries. The process of angiogenesis starts when several proangiogenic factors, such as vascular endothelial growth factor (VEGF) or basic fibroblast growth factor (bFGF), are excreted as a reaction to ischemia. These factors act by activating endothelial cells, causing them to proliferate and migrate into the perivascular space and eventually resulting in the formation of a new capillary lumen.

While angiogenesis is required for wound healing, increased angiogenesis is also associated with increased tumor growth and tumor metastasis [39, 80, 116]. This duality can also be in cardiovascular disease. Here, angiogenesis relieves ischemia by sprouting new capillaries [156] but also aggravates atherosclerosis by increasing plaque vascularization [102]. The functions of bFGF and VEGF are well known and often studied; however, other molecules, like CD40, seem to play crucial roles as well.

As described above, VEGF is a potent initiator of angiogenesis. Several studies have described a regulatory role for monocyte or macrophage CD40 in VEGF production. A study by Melter et al. shows that the treatment of HUVECs and monocytes with soluble CD40 ligand (sCD40L) results in an induction of VEGF and VEGF-mRNA. In an in vitro endothelial cell growth assay, CD40L induced marked growth of HUVECs. Neutralizing anti-VEGF antibody completely inhibited the effect of sCD40L on HUVEC growth. The study also showed a model of SCID mice bearing human skin transplants. sCD40L was injected into the human skin grafts and a marked induction in VEGF expression was found after 7 days in all sCD40L-treated skins compared with controls [95]. A similar study, performed for 6 weeks, showed similar results. In a model of SCID mice bearing human skin transplants, it was found that the injection of CD40L-expressing cells, but not control cells, resulted in the in vivo expression of several angiogenesis factors (including VEGF and fibroblast growth factor) and a marked angiogenesis reaction [121]. Yet, another study showed that MCP-1 and CD40L stimulation of macrophages had a synergistic effect on COX-2 expression and subsequent VEGF production in gastric cancer [44]. All these studies clearly suggest that CD40 ligation is a potent stimulator angiogenesis and that the proangiogenic effect of CD40 is VEGF dependent.

While not many studies describing angiogenesis and CD40 on macrophages have been done, from the few that have been done a clear picture emerges, macrophage CD40 ligation induces and controls VEGF and other angiogenic factors, and seems to be an important factor in angiogenesis.

## Arteriogenesis

Arteriogenesis refers to the widening of existing collateral arteries, as to increase blood flow.

These existing collateral arteries are present at birth and can be considered as alternative ways blood can flow from one point to another. Thus, when a vessel becomes partially obstructed, collateral vessels are somewhat able to compensate for the loss of blood flow. Arteriogenesis causes these collateral vessels to widen as to increase to blood flow to its original state.

The exact mechanisms of arteriogenesis have not yet been unraveled. Arteriogenesis is initiated by an increase in shear stress, caused by an increased blood flow in the collateral artery after partial obstruction of the main artery. The shear stress causes endothelial cells to become activated, and expresses several chemo-attractants and adhesion molecules [56]. These cause monocytes to adhere, migrate, and change into macrophages. These macrophages start to produce several cytokines and growth factors, which cause smooth muscle cells (SMCs) to proliferate, facilitating vascular luminal expansion and, thereby, an increased collateral perfusion.

It has been shown that having increased arteriogenesis, and thus, a better collateral network is highly beneficial. Patients with a better collateral network have smaller lesions after myocardial infarction, less ventricular aneurisms, a relatively better left ventricular ejection fraction [50], less future cardiovascular events [9], and an improved survival [52, 94].

High expression of several immune modulating proinflammatory agents, such as Interferon-beta and Galectin-2, were shown to correlate with poor collateralization in humans and to directly inhibit this process in murine models [126, 145, 158]. It has been shown that the balance between M1 and M2 macrophages is of importance in arteriogenesis. For example, in a mouse model by Takeda et al., skewing macrophages toward the M2-phenotype resulted in an increase in arteriogenesis and a marked decrease in ischemia as a result [23, 81, 134]. Although no direct studies on CD40 and arteriogenesis have been done, it has been convincingly shown that CD40 on macrophages might be involved. In a mouse model to test the effects of galectins on arteriogenesis, they show that galectin administration leads to increased numbers of CD40-positive M1 macrophages and reduced numbers of M2 macrophages surrounding actively remodeling collateral arteries [158]. Suggesting that, in concurrence with the data described above, the shift toward CD40 positive M1 macrophages slows down arteriogenesis. As described above in the data presented about atherosclerosis, interfering with CD40 macrophage signaling induces a phenotype shift from M1 to M2 macrophages. While not many evidence exist yet, this might yield interesting new therapies for ischemic diseases.

## Discussion

**Fig. 2:**
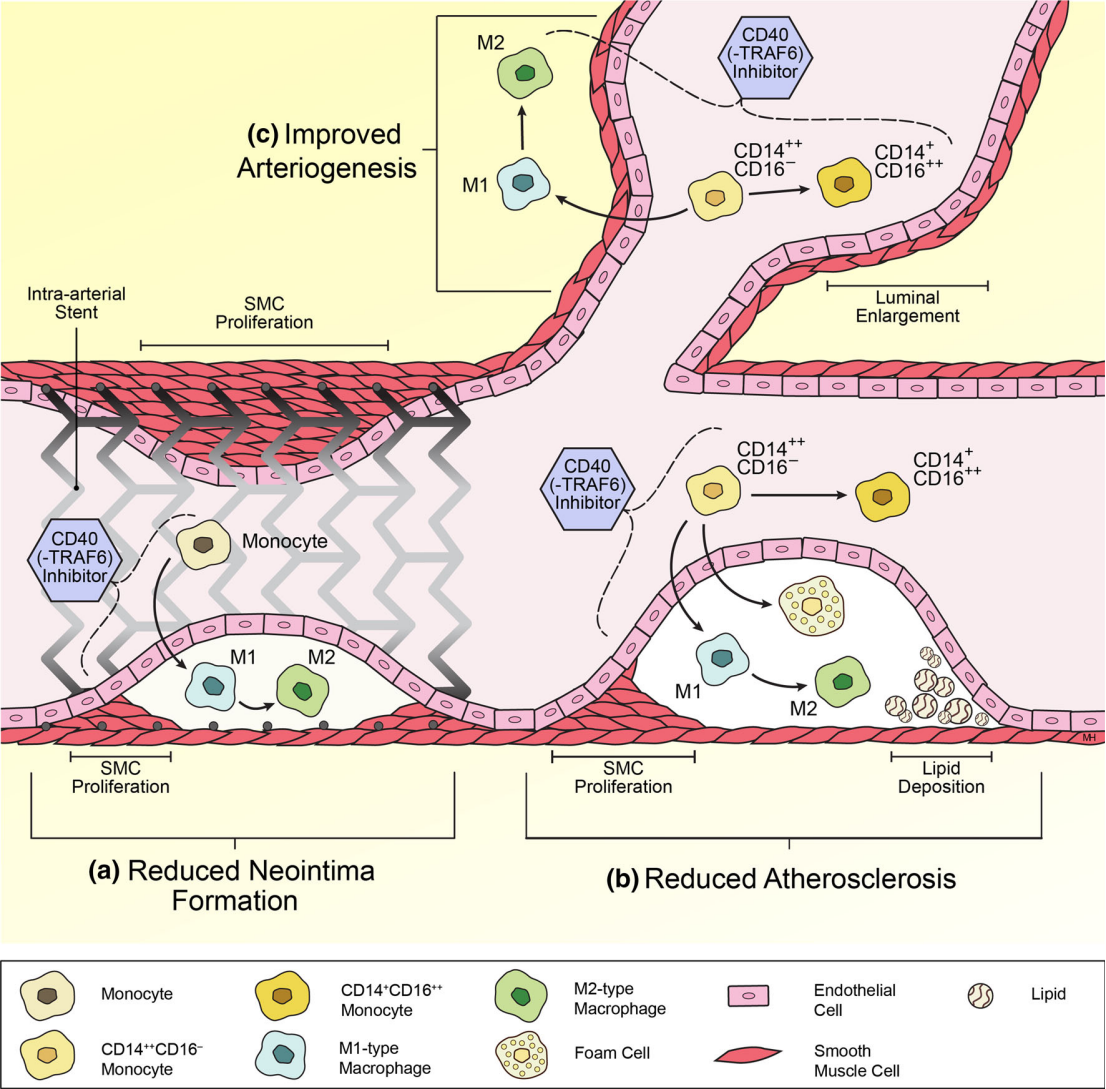
Overview of the proposed mechanisms of CD40 (-TRAF6) inhibition on ischemic heart diseases. **a** In neointima formation, CD40(-TRAF6) inhibition reduces monocyte/macrophage recruitment into the vessel wall and shifts the macrophage subset balance toward the regulatory M2 phenotype. CD40(-TRAF6) inhibition markedly reduces neointima formation. **b** In atherosclerosis, CD40(-TRAF6) inhibition induces Ly6C low monocytosis in mice (the human counterpart to Ly6C low is CD14+/CD16++). Furthermore, it reduces monocyte/macrophage recruitment into the plaque, shifts the macrophage subset balance toward the regulatory M2 phenotype and reduces the formation of foam cells. CD40(-TRAF6) inhibition abolishes atherosclerosis. **c** In arteriogenesis, CD40(-TRAF6) inhibition induces Ly6C low monocytosis in mice (the human counterpart to Ly6C low is CD14+/CD16++). Moreover, shifts the macrophage subset balance toward the regulatory M2 phenotype. Both these changes have been shown to be highly beneficial in arteriogenesis

Macrophages are one of the most versatile cells, and the CD40 receptor on these cells has an equally large range of functions. As described above, there is substantial evidence for a large role of CD40 on macrophages in ischemic heart disease. Evidence is presented that the activation of CD40 on macrophages accelerates atherosclerosis, accelerates neointima formation, and attenuates arteriogenesis. The downstream target of CD40, TRAF6, plays a major role in these processes. An overview of the proposed mechanisms of CD40 inhibition on ischemic heart diseases is shown in Fig. 2.

While much research still needs to be done, the data presented in this review suggest that interfering in CD40 signaling on the macrophage is an excellent candidate for future cardiovascular therapies. Targeting CD40 specifically on the macrophage can be done in several ways. Macrophage-specific CD40 can be inhibited using a bispecific antibody that binds to both CD40 and a macrophage specific receptor. However, a more practical way is to use liposomes or HDL nanoparticles containing a CD40(-TRAF6) inhibitor. These particles naturally target phagocytic cells, particularly macrophages, and thus specifically deliver the drugs to these cells [73, 125]. Theoretically, a patient’s ischemic burden could be drastically lowered by reducing or inhibiting CD40 signaling on macrophages. It could act as a “double-edged sword” by reducing the cause of ischemic heart disease (atherosclerosis) and promoting the cure (arteriogenesis). Equally important is the notion that reducing or inhibiting CD40 signaling on macrophages would not induce new problems, such as heightened tumor growth (induced by increased angiogenesis) or immune-suppression (by not interfering in the adaptive immune system). Although significant research is still required, CD40 on macrophages is an exciting and potent therapeutic target. A drug targeting this interaction could possibly lead to new therapies and improved care in cardiovascular disease.
